# EUS-Guided Ethanol Ablation of Insulinomas: Case Series and Literature Review

**DOI:** 10.1097/MD.0000000000000085

**Published:** 2014-09-19

**Authors:** Shan-yu Qin, Xiu-ping Lu, Hai-xing Jiang

**Affiliations:** Department of Gastroenterology (SQ, XL, HJ), First Affiliated Hospital of Guangxi Medical University, Nanning, Guangxi, China.

## Abstract

Surgical resection is a standard treatment for insulinomas; however, it is associated with a high risk of complications and limited to specific suitable candidates. In recent years, endoscopic ultrasound (EUS)-guided ethanol ablation of insulinomas has emerged as a new therapeutic option, especially for elderly patients and candidates unfit for surgery. We aimed to evaluate the feasibility and safety of this technique for insulinomas.

Four patients diagnosed with insulinomas based on EUS–fine-needle aspiration and immunohistochemistry results underwent EUS-guided 95% ethanol ablation. A comprehensive literature review was performed to understand the current status of the feasibility, safety, and effects of EUS-guided ethanol ablation of insulinomas.

EUS-guided ethanol ablation of insulinomas was successfully completed in all the 4 patients. There were no perioperative or postoperative complications. The patients were discharged at 3 days after the procedure. No recurrence of hypoglycemia or tumors was noted during follow-up (range, 3–6 months). Literature review showed 8 patients with insulinomas who underwent EUS-guided ethanol ablation. All the procedures were successful, with no need for further surgical treatment. Among these reviewed cases, 6 patients had no post-procedural complications, while other 2 patients showed a mild increase in the serum levels of lipase and/or pancreatic enzymes within 48 h post-procedure; furthermore, 1 of these 2 patients presented at a later date with medically controllable hematoma and ulceration. During follow-up, 6 patients remained asymptomatic and normoglycemic, while the 2 patients who presented post-procedural complications developed occasional mild confusion.

EUS-guided ethanol ablation of insulinomas is an effective and safe modality, with an acceptable level of post-procedural complications. However, the long-term effects of this new therapeutic option need to be validated in a large randomized controlled trial with longer follow-up.

## INTRODUCTION

Insulinoma—a type of neuroendocrine tumor (NET)—is characterized by hypoglycemic symptoms and secretion of abnormal endogenous insulin. The annual incidence of insulinomas in the general population is approximately 1 to 4 per million; however, it has been reported to be higher in autopsy studies (0.8%–10%), indicating that this tumor is often undiagnosed.^1^ Surgical enucleation or resection of the insulinoma is considered to be the standard treatment at present; however, resection is associated with high mortality and morbidity, being 0% to 4% and 10% to 43%, respectively.^2–4^ In addition, surgical treatment is unsuitable for elderly patients or patients with poor general condition who are unfit for surgery. In recent years, endoscopic ultrasound (EUS)-guided ethanol ablation of insulinomas has emerged as an alternative minimally invasive therapy and has been attempted in elderly patients, patients in whom surgery was not feasible, and patients who refused surgical treatment.^4,5^ However, to date, little data is available regarding the efficacy of this technique for insulinomas. In this report, we describe 4 cases of EUS-guided ethanol ablation of insulinomas and review related studies in order to evaluate the feasibility, safety, and efficacy of this technique.

## PATIENTS AND METHODS

### Patient and Procedures

Four patients who were unwilling to accept surgical resection for insulinomas were enrolled in the First Affiliated Hospital of Guangxi Medical University, Guangxi, China, between November 2013 and January 2014. The study was approved by the Institutional Review Board of Guangxi Medical University. All the enrolled patients provided informed consent for the procedure. Coagulopathy was excluded in each patient prior to the procedure. EUS–fine-needle aspiration (FNA) procedures were performed by an expert endosonographer (S.Q., with 8 years of EUS–FNA operative experience). The patients were diagnosed by using EUS–FNA, with the aspirated materials examined by smear cytology and immunohistochemistry; after confirming the diagnosis, they underwent EUS-guided ethanol ablation.

### EUS–FNA Procedures

EUS was performed by linear array echoendoscope (Olympus Ltd, Tokyo, Japan). FNA was performed using a 22-gauge needle (Wilson Cook Medical, Winston-Salem, NC). A transduodenal approach was used for pancreatic head lesions, and a transgastric approach was used for lesions in the pancreatic body or tail. Aspirated materials were expelled onto a glass slide and fixed with appropriate manipulations by skillful cytotechnicians. Slides were transported to the laboratory as soon as the procedure was completed. Three immunohistochemical indices were tested, including creatine kinase, chromogranin A (CgA), and spiral ganglion neurons, which were used for confirming the diagnosis of insulinoma. We used the 2010 WHO classification for NETs, that is, tumors were graded as G1 (well-differentiated NETs), G2 (well-differentiated carcinoma), and G3 (poorly differentiated neuroendocrine carcinoma).^6^ Mitotic counts and the Ki-67 index were used to grade NETs, with the mitotic count in G2 NET being between 2 and 20 per high-power field and a Ki-67 index between 3% and 20%, while the values for G1 and G3 NET were lower and higher, respectively, than those for G2 NET.^6^

### EUS-Guided Ethanol Ablation Technique

EUS-guided ethanol ablation was performed by using a linear echoendoscope (GF-UC140P-AL5 or GF-UC160P-AT8; Olympus America Inc, Center Valley, PA) with a 25-gauge needle (Wilson Cook Medical). The volume of the 95% ethanol injection was calculated according to the size of the tumor. In our center, the volume was calculated as follows. For round tumors, the volume of ethanol injection was half of the size of the tumor, and for oval or irregular tumors, the volume of ethanol injection was calculated according to the following formula: (major axis + minor axis of the tumor)/2. When the tumor was located close to a vessel or the pancreatic duct, the volume of ethanol injection was reduced to half or one third of the normal injection volume. Precise injection was possible with the use of a 1.0-mL syringe. We attempted to complete all the injections in a single treatment session in order to minimize possible complications.

We slowly advanced the needle into the center of the tumor and injected small aliquots of 95% ethanol, typically 0.01 to 0.1 mL at a time. The injections were repeated at the same site until a hyperechoic blush was seen expanding in the tumor on ultrasound. Injections within a particular site were terminated when the hyperechoic blush extended in close proximity to the edge of the tumor or when there is a concern of leakage beyond the tumor border (close proximity to vessels and other structures). When the needle was being withdrawn, additional small injections were administered until the needle was nearly completely removed. Before removal, the needle was held in place for approximately 1 minute to minimize the tracking of ethanol into adjacent structures. Based on the tumor size and pattern of spread after the initial injection, additional passes were made, avoiding the previous needle tracts. The patient was maintained nil by mouth for 1 day after the procedure, and blood glucose levels were monitored for 3 days postoperatively. The patient was discharged when the blood glucose level increased. The patients were followed up at 1 month, 6 months, and 1 year after the procedure, with a further follow-up scheduled at 2 years after the procedure. Besides EUS test, the patients were also given enhanced computed tomography (CT) to evaluate the change of pancreatic lesion. Furthermore, patients were to be hospitalized in case of any recurrence of hypoglycemia. Fasting blood glucose (FBG), C-peptide levels, CgA levels, and insulin release index were tested during each follow-up.

## RESULTS

All the 4 patients presented typical Whipple’s triad at the time of diagnosis, and the symptoms of hypoglycemia, such as confusion, disturbance of consciousness, and abnormal behavior, were onset at the morning. The FBG ranged from 1.7 to 2.8 mmol/L and the mean value was 2.1 mmol/L. The peripheral blood insulin/glucose ratio was >0.4. The initial diagnosis of these patients was skeptical of insulinoma. After ethanol ablation, the symptoms of insulinomas showed significant amelioration at 1 day postoperatively in 3 patients, and the mean FBG increased to 3.7 mmol/L at the first day after treatment; in the remaining patients, amelioration was observed on the second day postoperatively, and the FBG increased to 3.5 mmol/L. The changes in FBG, C-peptide level, CgA levels, and insulin release index suggested increased improvement in the patients’ condition. The data for the 4 patients are shown in Table 1.

**TABLE 1:**
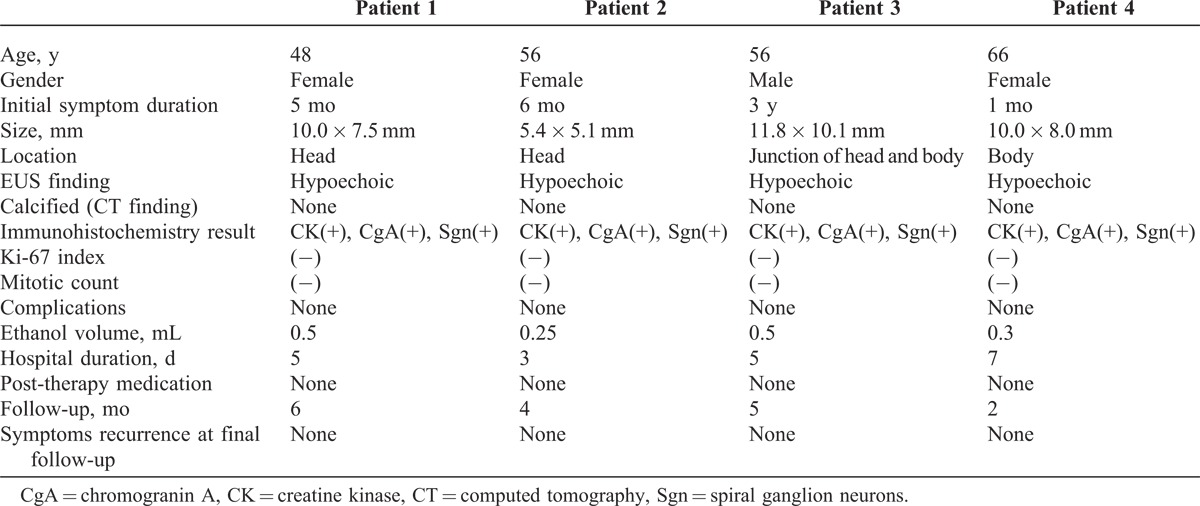
Data of Four Patients of Insulinomas

**FIGURE 1:**
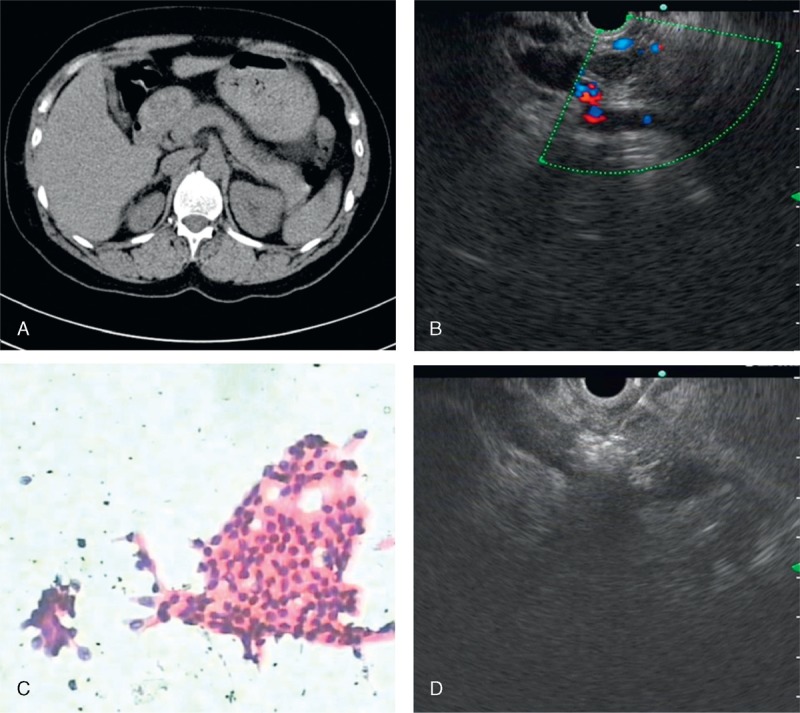
(A) CT showing no lesion in the pancreas. (B) EUS confirming the presence of a hypoecho and uniform lesions in the head of pancreas with 10 × 7.5 mm in size, adjacent to the porta vein and gastroduodenal artery. (C) EUS-FNA yielded cytology (Diff Quick, ×20) compatible with a neuroendocrine tumor, immunohistochemical result showed that heterocyst CK(+), CgA(+), Sgn(+). (D) A-22 gauge needle was inserted and a total of 0.5ml of 95% ethanol was injected in the lesion, the ethanol produced a hyperechoic infiltrate. CgA = chromogranin A, CK = creatine kinase, CT = computed tomography, EUS = endoscopic ultrasound, FNA = fine-needle aspiration.

In all the 4 patients enrolled in this study, the insulinomas were benign and were confirmed by cytopathology and immunohistochemistry (Figure 1) by using EUS-FNA. However, one of the insulinomas failed to be detected by CT and magnetic resonance imaging (MRI), suggesting the high diagnostic accuracy and safety of EUS–FNA. The insulinomas were all graded as G1 NET, based on the Ki-67 index and mitotic count. All the procedures were successfully completed in a single setting without any complications. The mean volume of ethanol injection was 0.39 ± 0.13 mL. All the patients were discharged at 3 days after the procedure. During the follow-up (range, 3–6 months), no recurrence of hypoglycemic symptoms was noted, none of the patients required medical therapy, and no later-onset complications occurred.

We also performed a systematic review by searching electronic databases (PubMed, Web of Science, MEDLINE, Cochrane Library, and Google Scholar) prior to March 2014 without limitation of the language. In literature review, 4 studies^2,4,7,8^ reported the efficacy of EUS-guided ethanol ablation on 8 patients with insulinomas. The concentrations of ethanol used ranged from 95% to 99%. Most lesions ranged in the size from 1 to 2 cm in diameter, and the maximum lesion measured 20 × 18 mm in size. Two studies provided the pathological diagnosis by EUS–FNA before ethanol injection. In these studies, patients had refused surgery because of poor general condition, such as concurrent hypertension or severe aortic stenosis, which posed a high risk of surgical complications with advanced age. No operative complications were reported in any study. Six patients showed no complications postoperatively; however, the other 2 patients showed a mild increase in the serum levels of lipase and/or pancreatic enzymes in 48 h postoperatively, with later occurrence of medically controlled hematoma and ulceration. During follow-up (the longest period >34 months), 6 patients remained asymptomatic and normoglycemic, while the 2 patients who presented post-procedural complications developed occasional mild confusion.

## DISCUSSION

Insulinoma typically leads to symptomatic fasting hypoglycemia. Once insulinoma is considered as a diagnosis, patients should undergo preoperative localization. However, most insulinomas measure <2 cm or even <1 cm; thus, advanced imaging technology is required for these tumors. Noninvasive imaging techniques such as CT, MRI, and ultrasonography can localize tumors that are >1 cm but fail to capture small insulinomas. EUS–FNA is an invasive technique but has a high diagnostic accuracy, and the aspirated materials obtained by EUS–FNA can be tested by cytopathology and immunohistochemistry, which is helpful for differentiating between benign and malignant tumors. In the present study, 4 patients of insulinomas were diagnosed by EUS–FNA, with no complications associated with the procedure, while 1 patient with insulinomas failed to be detected by CT and MRI, suggesting the high diagnostic accuracy and safety of EUS–FNA.

Surgery is the standard treatment for insulinomas, with an extremely high success rate. Although the postoperative mortality associated with surgical resection for insulinomas has reduced over the last 2 decades, the morbidity continues to be high.^9^ Common postoperative complications include pancreatic leakage, pancreatitis, hemorrhage, pseudocyst, intra-abdominal abscess, and diabetes. Medical treatment is also available to those who are unable or unwilling to undergo surgical treatment, for preoperative control of blood glucose levels or for unresectable metastatic disease. However, studies have indicated that these drugs are not effective, have various side effects, and enable long-term control only in a minority of patients.^10,11^

Ethanol is a particularly effective ablative agent, being cost-effective and rapid in action. Ethanol injection had been successfully used for the ablation of thyroid, spleen, and liver tissue as well as renal cysts, with minimal side effects.^12,13^ Previously, EUS-guided ethanol ablation therapy for pancreatic lesions has been reported in some centers. A multicenter, randomized, prospective trial indicated that EUS-guided 80% ethanol injection resulted in a greater decrease in the size of pancreatic cystic tumors than saline solution injection.^14^ Dewitt et al^15^ described 9 patients with pancreatic cysts who were followed-up for 2 years after EUS-guided ethanol ablation, with no evidence of cyst recurrence. In our center, we have also successfully performed EUS-guided ethanol ablation for several metastatic lesions located at the liver or the pancreas, and found that this therapeutic strategy can relieve symptoms and/or prolong survival. Thus, we consider that if the pathological results confirm the presence of a benign insulinoma, EUS-guided ethanol ablation of such insulinomas may be an alternative therapeutic technique to surgical resection, given that there are no contraindications to the endoscopic procedure, regardless of the patients’ age or the size of the lesion.

The technique of EUS-guided ethanol ablation of insulinomas has emerged only in recent years. According to our literature review, all the patients who underwent this therapy were elderly, were considered unfit for surgery, or refused surgery. Jurgensen et al^7^first reported EUS-guided ethanol ablation of an insulinoma in a 78-year-old woman with poor general condition who refused surgery. Postoperatively, the patient showed symptomatic improvement, with no recurrence of the tumor on follow-up. Levy et al^4^ reported the largest case series (5 patients) for EUS-guided ethanol ablation of insulinomas; they observed that the hypoglycemic symptoms of insulinomas were relieved almost immediately after the procedure and that this symptomatic relief was maintained during the follow-up (range, 5–38 months). Our study was the second largest case series (4 patients) for this technique; we noted no postoperative complications, a shorter hospital stay, and no recurrence of hypoglycemia during follow-up. These results indicate the effectiveness and safety of EUS-guided ethanol ablation of insulinomas.

As a new treatment, in addition to its clinical efficacy, EUS-guided ethanol ablation of insulinomas has various advantages in terms of patient selection, minimal invasiveness, shorter hospital stay, and a lower risk of post-procedural complications than surgical treatment. Moreover, this technique can be performed repeatedly and on different locations of a lesion; furthermore, it is associated with fewer contraindications and complications. Therefore, we consider it reasonable to hypothesis that EUS-guided ethanol ablation is also useful for treating metastatic lesions located at the liver or the pancreas, which may improve symptoms or prolong the survival for these patients.

Despite its advantages, this technique does require further study. With respect to the complications associated with EUS-guided ethanol ablation of insulinomas, previous studies^2,4,7,8^ have reported localized pain in the upper abdomen or a mild elevation of amylase and lipase levels as post-procedural complications; however, these symptoms typically subsided after 48 h. Rare complications include mild confusion and later occurrence of medically controlled hematoma and ulceration of the duodenal wall.^2^ The reports explain that the causes of the complications are mainly related to the experience of the operator. With regard to the volume and concentration of ethanol injection, there is no agreement at present. The concentration and the volume of ethanol widely varies (range, 95%–98%), even in the same center. Levy et al^4^ described that they injected as much ethanol as possible to infiltrate the entire tumor; however, their aim was symptomatic relief rather than complete ablation of the tumor, thus, lower volumes of ethanol and repeated treatment sessions may have been more appropriate. In our center, based on our experience, we consider that to treat insulinomas effectively and safely, the volume of ethanol injected in a single treatment session should not be greater than half the volume of the tumor; repeat injections can be administered if and when necessary.

There are certain limitations for EUS-guided ethanol ablation of insulinoma. First, when the lesion is small or located close to blood vessels, it is difficult to perform ethanol ablation; furthermore, the technique poses a high risk of hemorrhage. This may, eventually, lead to surgical treatment. Second, no studies have thus far reported whether this technique is appropriate for multiple insulinomas in the same patient. Third, little data is available regarding the efficacy of this technique for insulinomas with no multicenter controlled studies; current studies mostly comprise case reports or case series. Therefore, further controlled studies are essential for verifying the efficacy of this technique for insulinomas.

In summary, our results and a review of the literature demonstrated that EUS-guided ethanol ablation of a single, small insulinoma is feasible, minimally invasive, safe, and effective, with an acceptable level of post-procedural complications. This new technique might, therefore, be applied to a wider range of potential candidates with poor general condition or those refusing surgical treatment.
